# Targeted Re-Sequencing of the 2p21 Locus Identifies Non-Syndromic Cleft Lip Only Novel Susceptibility Gene *ZFP36L2*

**DOI:** 10.3389/fgene.2022.802229

**Published:** 2022-02-09

**Authors:** Mu-Jia Li, Jia-Yu Shi, Qiu-Shuang Zhu, Bing Shi, Zhong-Lin Jia

**Affiliations:** ^1^ State Key Laboratory of Oral Diseases and National Clinical Research Center for Oral Diseases, Department of Cleft Lip and Palate, West China School of Stomatology, Sichuan University, Chengdu, China; ^2^ Division of Growth and Development and Section of Orthodontics, School of Dentistry, University of California, Los Angeles, Los Angeles, CA, United States

**Keywords:** 2p21, non-syndromic cleft lip only, ZFP36L2, proliferation, cell cycle

## Abstract

rs7590268 present on the 2p21 locus was identified to be associated with non-syndromic cleft lip with or without cleft palate (NSCL/P) in several populations, including the Chinese Han population, indicating that 2p21 was a susceptibility locus for NSCL/P. However, previous studies have only identified common single-nucleotide polymorphism (SNP) within the *THADA* gene, neglecting the rare variants and other genes in 2p21; thus, this study was designed to investigate additional variants and novel susceptibility genes in 2p21. A total of 159 NSCL/P patients and 542 controls were recruited in the discovery phase, whereas 1830 NSCL/P patients and 2,436 controls were recruited in the replication phase. After targeted region sequencing, we performed association and burden analyses for the common and rare variants, respectively. Furthermore, RNA-seq, proliferation assay and cell cycle analysis were performed to clarify the possible function of the candidate gene *ZFP36L2*. Association analysis showed that four SNPs were specifically associated with non-syndromic cleft lip only (NSCLO) and two SNPs were associated with both NSCLO and NSCL/P. Burden analysis indicated that *ZFP36L2* was associated with NSCLO (*p* = .0489, OR = 2.41, 95% CI: 0.98–5.90). Moreover, SNPs in the *ZFP36L2* targeted gene *JUP* were also associated with NSCLO. *ZFP36L2* also inhibited cell proliferation and induced G2 phase arrest in the GMSM-K cell line. Therefore, we proposed that *ZFP36L2* is a novel susceptibility gene of NSCLO in the 2p21 locus, which could lead to NSCLO by modulating cell proliferation and cycle.

## Introduction

Non-syndromic orofacial clefts (NSOFCs), which usually occur without any other physiological abnormalities, are one of the most common birth defects. NSOFCs are commonly divided into non-syndromic cleft lip with or without cleft palate (NSCL/P) and non-syndromic cleft palate only (NSCPO), which have been historically regarded as etiologically distinct phenotypes, because they differ in epidemiology and family patterns, as well as in the developmental origin of the lip and palate (Marazita, 2012). NSCL/P, on the other hand, includes two phenotypes: non-syndromic cleft lip only (NSCLO) and non-syndromic cleft lip with palate (NSCLP), but they are usually grouped together and considered as the same defect with different severity (Mitchell et al., 2002); however, some researchers have suggested that NSCLP and NSCLO may also be etiologically distinct, and should be analyzed separately when possible (Harville et al., 2005; Sivertsen et al., 2008; Grosen et al., 2010).

Individuals with NSCL/P are usually exposed to a series of problems early in life, such as difficulties in feeding and ear infections, which impose a heavy burden on both the affected families and society. Additionally, NSCL/P impact the patients’ quality of life throughout their life span, although surgical repair, speech therapy, dental care, and psychological support are available (Wehby and Cassell, 2010). Therefore, it is of great significance to clarify the pathogenesis of NSCL/P. Currently, there is a consensus that genes, environmental factors, and their complicated interactions contribute to the occurrence of NSCL/P (Machado et al., 2016).

In the past few decades, several environmental factors have been identified to be associated with NSCL/P, including smoking, drinking, drug consumption (such as antiepileptic agents), and lack of dietary folic acid during early pregnancy (Honein et al., 2007; Stott-Miller et al., 2010). Although a variety of methods have been used to identify its susceptibility gene, progress toward its identification has been slow. Genome-wide association studies (GWASs) have been an effective tool in identifying genome variants associated with NSCL/P, aiding identification of over 40 susceptibility loci in the past few years (Birnbaum et al., 2009; Mangold et al., 2010; Ludwig et al., 2012; Sun et al., 2015).

Based on the first meta-analyses of NSCL/P, which combined the data from two large GWAS, including 666 European trios and 795 Asian trios, 2p21 was proposed to be susceptibility region for NSCL/P for the first time (Ludwig et al., 2012). Subsequent studies conducted in different populations further proved that variants in 2p21 locus were associated with NSCL/P; among them, rs7590268 was identified several times in the European population (Beaty et al., 2013; Moreno Uribe et al., 2017). Replication studies showed that rs7590268 also had a strong signal in the southern Chinese population (Pan et al., 2013).

These findings implied that 2p21, where rs7590268 was located, was a susceptibility locus for NSCL/P. However, genotyping arrays used in GWAS only capture 5% of the total SNPs occurring genome wide, potentially missing causal SNPs that are in linkage disequilibrium with SNPs captured in GWAS; and, GWAS neglects rare variants, defined here as minor allele frequency (MAF) < 0.01, which have a larger effect than common variants and could partially compensate for the missing heritability (Manolio et al., 2009; Tada et al., 2016; Sazonovs and Barrett, 2018). Additionally, the reported SNPs were mainly located in the *THADA* gene. Although *THADA* did not involve in craniofacial development according to the currently published literature, variations within it have been associated with many diseases, which may be attributable to its large size (370 kb), the effect of regulatory elements, or the other adjacent genes (Drieschner et al., 2007; Ludwig et al., 2012). Notably, a recent study proposed another gene—*ZFP36L2*—adjacent to *THADA*, as the lead risk NSCL/P gene in 2p21 for the first time (Lin-Shiao et al., 2019); however, neither common nor rare variants in the *ZFP36L2* gene have been identified.

ZFP36L2 belongs to the zinc finger protein ZFP36 family and is classified as a Cys–Cys–Cys–His (CCCH)-type zinc finger tandem protein (Ramos et al., 2004; Stumpo et al., 2009). It is well-known as an RNA-binding protein, which destabilizes target mRNA and, thus, influences the targeted gene expression by binding to AU-rich elements (ARE) in the 3′ untranslated region (UTR) of labile mRNAs(Lai et al., 2000). The function of specific ARE-binding proteins could be modulated by post-translational epigenetic modifications including methylation and phosphorylation (Galloway et al., 2016). When stimulated by lipopolysaccharide, *Zfp36l2* phosphorylation influences the production of inflammatory mediators by regulating Mitogen-activated protein kinase (Mkp)-1 mRNA expression (K. T. Wang et al., 2015). Additionally, *ZFP36L2* also participates in the epigenetic modification process. In the absence of *Zfp36l2*, oocytes failed to accumulate histone methylation at H3K4 and H3K9, leading to transcriptional silence (Dumdie et al., 2018). Furthermore, *Zfp36l2* could act as a safeguard against chromosomal instability and post-transcription replication stress during thymopoiesis (Vogel et al, 2016).

**FIGURE 1:**
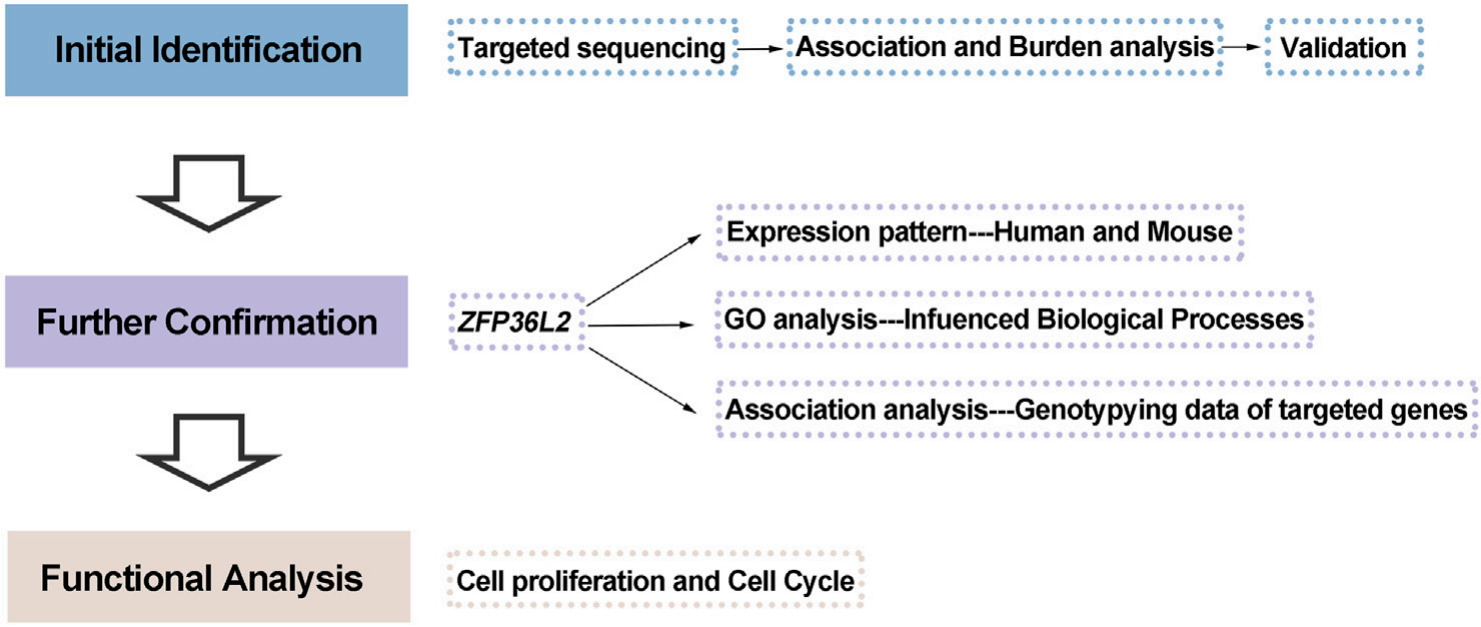
Details of the study design.

In the present study, we aimed to perform a comprehensive screening of susceptibility variants (both common and rare) in the 2p21 locus by targeted sequencing, followed by interpretation *via* association analysis, burden analysis, and a series of functional analyses (the study design is shown in Figure 1).

## Material and Methods

### Subject Characterization and Ethics Statement

In this study, we performed a two-phase case-control analysis, including an initial discovery phase and a subsequent replication phase. In the discovery phase, 159 unrelated patients with NSCL/P (80 NSCLP and 79 NSCLO patients) were selected from the Cleft Surgery Department of the West China College of Stomatology, Sichuan University. All of them self-identified as Han Chinese, and they did not have any other congenital anomalies. The whole-genome sequencing data of 542 Han Chinese normal controls (sequenced using Illumina Hiseq platform with an average coverage of 39.89) included in this phase were obtained from the Novogene internal database (http://www.novogene.com/).

The genotyping data of 1,626 patients with NSCL/P (including 579 patients with NSCLP, 1,047 patients with NSCLO) and 2,255 normal controls from two GWAS (Sun et al., 2015; Huang et al., 2019), as well as another 204 patients with NSCLO and 181 normal controls selected from the Cleft Surgery Department of West China College of Stomatology, Sichuan University, were recruited in the replication phase for inclusion in the study (Supplementary Table S1).

Human subject study protocols were approved by the Hospital Ethics Committee of West China Hospital of Stomatology, Sichuan University; these protocols conformed to the Strengthening the Reporting of Observational Studies in Epidemiology guidelines. Written informed consent was obtained from recruited individuals of consenting age and from parents on behalf of their participating children (WCHSIRB-D-2016-012R1).

### DNA Extraction and Quality Control

Peripheral blood samples were collected from all the participants and their parents, from which DNA was extracted using the salting out method and then stored in Tris-EDTA buffer. The quality of the isolated genomic DNA was verified by electrophoresis on 1% agarose gel to exclude the possibility of DNA degradation or RNA/protein contamination. Furthermore, the DNA purity and concentration were detected using a NanoPhotometer^®^ spectrophotometer (IMPLEN, CA, United States), with good quality output of ratio of optical density at 260 nm to the optical density at 280 nm (OD260/OD280) values ranging from 1.8 to 2.0.

### Selection of Targeted Region and Sequencing

According to the linkage disequilibrium (LD) structure shown by the CHB/JPT HapMap, the targeted region for deep sequencing was around rs7590268, based on ranges from chr2:43,417,119 to 43,838,705 (GRCh37/hg19), including exons, introns, and the intergenic region (Supplementary Figure S1). Sequencing was efficiently carried out using 1.0 μg genomic DNA in an Agilent liquid capture system (Agilent SureSelectXT Custom Kit) according to the manufacturer’s protocol. The DNA library was sequenced on Illumina Hiseq for paired-end 150 bp reads.

### Bioinformatics and Statistical Analysis

After quality control processing, including filtering of adapter-related reads, reads containing N, and low-quality reads, the clean sequence data were mapped to the GRCh37/hg19 human genome using Burrows-Wheeler Aligner (BWA) software (Li and Durbin, 2009). Then, SNVs and indels were identified by Sequence Alignment Map (SAM tools) (Li et al., 2009) and merged by VCF (Variant Call Format) tools (version 0.1.13) (Danecek et al., 2011). Later, variants were annotated by ANNOVAR (version 201707) (K. Wang et al., 2010), followed by function prediction via SIFT (Ng and Henikoff, 2003), v1.3 CADD (Rentzsch et al., 2018), Polyphen-2 (http://genetics.bwh.harvard.edu/pph2/) (Adzhubei et al., 2013) and Mutation Taster (http://www.mutationtaster.org/) (Schwarz et al., 2010).

Variants with call rates >95% were divided into two groups: common variants (MAF ≥0.01) and rare variants (MAF <0.01). Furthermore, we performed the Hardy-Weinberg equilibrium (HWE) test at each SNP. SNPs which deviated from HWE (*p* < .000001) were removed from subsequent association analysis, which was conducted by PLINK (version 1.9) (Purcell et al., 2007). The *p* value in the replication phase was adjusted for multiple corrections. Using the R package SKAT, gene-based burden analysis was performed on rare variants in accordance with the following criteria: 1) MAF <0.01 in CHB and CHS (CHB, Han Chinese in Beijing; CHS, Southern Han Chinese) from the 1,000 Genome database and Novogene internal database (http://www.novogene.com/); 2) MAF <0.001 in the Genome Aggregation Database (GnomAD); and 3) at least two prediction tools indicate the rare variation to be damaging (PolyPhen-2, SIFT, MutationTaster, CADD), the thresholds for damaging in each tool were set as follows: SIFT scores <0.05, Polyphen2_HDIV scores ≥0.957, Mutation Taster indicates “Disease causing”, CADD scores >10. Fisher’s exact test or Pearson chi-square test was performed between the cases and controls.

### Cell Culture, Transient Knockdown, and Overexpression

Considering the important role of oral epithelium in facial morphogenesis, as well as its known association with NSCL/P, a human oral epithelial cell line (GMSM-K, kindly gifted by Dr. Zhang from Peking University) was selected for functional analysis in our study (H. Liu et al., 2020; H. Liu et al., 2017). GMSM-K was cultured in Dulbecco’s modified Eagle’s medium supplemented with 10% fetal bovine serum (Gibco, United States) and 1% penicillin-streptomycin solution (Gibco, United States).

Small interfering RNA (siRNA) targeting *ZFP36L2* (NM_006887.4) (siRNA-*ZFP36L2*: 5′-GCC​UUC​UAC​GAU​GUC​GAC​UTT-3′) and negative control siRNA (siRNA-negative control: 5′-UUC​UCC​GAA​CGU​GUC​ACG​UTT-3′) were both designed and synthesized by GenePharma (Shanghai, China). Moreover, *ZFP36L2* was cloned into the pcDNA3.1 overexpression plasmid (YouBio, China) (Supplementary Figure S2), and the pcDNA3.1 plasmid without additional sequence served as a negative control. Both siRNAs and plasmids were transfected into GMSM-K using the SF Cell Line 4D X Kit S (Lonza, Germany) according to the manufacturer’s instructions.

### RNA-Seq, Differential Expression Analysis and Gene Ontology Analysis

GMSM-K cells were transfected with siRNA-negative control or siRNAs- *ZFP36L2* for 48 h. Then, the cells were collected and RNA-seq was performed using the BGISEQ-500 platform (BGI, China). Two biological replicates were included within each group. Differential gene expression analysis was performed using DEseq2 method (|log2| ≥ 0.8, Q ≤ 0.05), and GO enrichment analysis was performed on DEGs using DAVID 6.8 (Huang da et al., 2009).

### Quantitative Real-Time PCR Analysis

RNA was extracted using a Tissue/Cell RNA extraction Kit (Biobase, China) 48 h after transfection, and was then reverse-transcribed to cDNA using PrimeScript™ RT reagent Kit (Takara Biotechnology, Dalian, China). RT-qPCR was performed using TB Green^®^ Premix Ex Taq™ (Takara Biotechnology, Dalian, China) on a LightCycler 480 System (Roche, Switzerland). All experiments were performed in triplicate at least, each with three technical replicates. The results were calculated using equation 2^−ΔΔCt^. The primers used are shown in Supplementary Table S2.

### Proliferation Assay

Upon transfection, GMSM-K cells were seeded into 96-well plates at a density of 2 × 10^4^ cells/100 μl. After 24, 48, and 72 h, 10 μl of Cell Counting Kit-8 (CCK-8) (APExBIO, United States) was added to each well. Then, 3 h later, OD of each well was measured at a 450 nm wavelength. Measurements at each time point were replicated three times having five replicates each. Results are shown as mean ± SEM. Statistical analysis was performed using the unpaired two-tailed *t*-test in GraphPad Prism eight software.

### Cell Cycle Analysis

Approximately 2 × 10^5^ transfected GMSM-K cells were plated in 6-well plates. After 48 h, cells were collected for further cell cycle analysis, in which cells were fixed in 70% ethanol at 4°C overnight, washed twice with cold phosphate buffer saline, and re-suspended in 0.5 ml PI staining reagent (25 μl propidium iodide (20X), 10 μl RNase A (50X), and 0.5 ml sodium citrate buffer) (Cell Cycle and Apoptosis Analysis Kit, Beyotime, China). The cells were then incubated in the dark at 37°C for 0.5 h. Samples were detected using an Attune NxT flow cytometer (Thermo Fisher, United States). FCS files were downloaded and analyzed using FlowJo software (version 10.4). For each group, results are shown as the mean ± SEM of three replicates. Statistical analysis was performed using the unpaired two-tailed *t*-test in GraphPad Prism eight software.

## Results

In the discovery phase, from the 159 NSCL/P cases, we identified a total of 2,352 Single-nucleotide variants (SNVs) and 552 insertion/deletions (indels), including variants located in exons, splice sites, introns, UTRs, and intergenic regions (Supplementary Figure S3). For common and rare variants that met the inclusion criteria, we performed case-control association analysis and burden analysis among the 159 NSCL/P cases and 542 normal controls, respectively.

### SNPs Within 2p21 Locus Were Significantly Associated With NSCLO

A total of 312 common SNPs (MAF ≥0.01) that passed the threshold of HWE (*p* > .000001) were recruited for association analysis. When compared with the *p* value 1.60E-04, which was adjusted for multiple corrections (*p* = .05/312), only rs201795193 (*p* = 2.12E-08, OR = 0.33, 95% CI: 0.21–0.52) was significantly associated with NSCL/P.

Seven SNPs (rs201795193, rs12990267, rs199721109, rs74343467, rs13002812, rs6544660, and rs12478601) with *p* value less than .05 were replicated in larger cohorts (1,626 patients with NSCL/P and 2,255 normal controls) to test their associations with NSCL/P, NSCLP, and NSCLO (Table1, Supplementary Table S3). Six out of seven SNPs were significantly associated with NSCLO after multiple corrections (*p* = .05/7 = 7.14E-03). Among these, four SNPs, including rs201795193 (*p* = 1.62E-03, OR = 0.78, 95% CI: 0.67–0.91), rs12990267 (*p* = 5.09E-04, OR = 0.76, 95% CI: 0.65–0.89), rs199721109 (*p* = 5.77E-04, OR = 0.76, 95% CI: 0.65–0.89), and rs74343467 (*p* = 1.86E-03, OR = 0.79, 95% CI: 0.69–0.92) were specifically associated with NSCLO; the other two SNPs, rs6544660 and rs12478601, were associated with both NSCLO (rs6544660: *p* = 5.21E-04, OR = 0.77, 95% CI: 0.67–0.89; rs12478601: *p* = 7.47E-04, OR = 0.78, 95% CI: 0.67–0.90) and NSCL/P (rs6544660: *p* = 2.78E-03, OR = 0.84, 95% CI: 0.74–0.94; rs12478601: *p* = 6.89E-03, OR = 0.85, 95% CI: 0.76–0.96), where the significant signal of NSCL/P might be driven by the NSCLO since both SNPs show lower *p* values in NSCLO than that in NSCL/P (Table 1). All the six significantly associated SNPs are novel, and we proposed 2p21 as a susceptibility locus for NSCLO in the Han Chinese population for the first time.

**TABLE 1:** Allelic association analysis for the SNPs in 2p21 locus.

SNP Position (hg19)	Discovery phase^a^					Replication phase^b^										
	Alt/Ref allele	MAF		OR (95%CI)	*p*-value	Minor/Major allele	MAF				NSCL/P		NSCLP		NSCLO	
		NSCL/P	Control				NSCL/P	NSCLP	NSCLO	Control	OR (95%CI)	*p*-value	OR (95%CI)	*p*-value	OR (95%CI)	*p*-value
rs201795193	T/C	0.05	0.16	0.26	2.12E-08	T/C	0.18	0.19	0.16	0.24	0.86	1.50E-02	0.93	4.12E-01	0.78	1.62E-03
chr2:43771991				(0.15, 0.45)							(0.75, 0.97)		(0.78, 1.11)		(0.67, 0.91)	
rs12990267	G/C	0.01	0.04	0.24	6.18E-03	G/C	0.19	0.20	0.16	0.24	0.84	7.22E-03	0.93	3.76E-01	0.76	5.09E-04
chr2:43770432				(0.07, 0.77)							(0.74, 0.95)		(0.78, 1.10)		(0.65, 0.89)	
rs199721109	G/A	0.16	0.22	0.68	1.90E-02	G/A	0.19	0.20	0.16	0.24	0.84	7.52E-03	0.92	3.71E-01	0.76	5.77E-04
chr2:43772391				(0.49, 0.94)							(0.75, 0.96)		(0.78, 1.10)		(0.65, 0.89)	
rs74343467	T/C	0.19	0.25	0.69	2.01E-02	T/C	0.25	0.25	0.23	0.28	0.86	1.52E-02	0.93	3.81E-01	0.79	1.86E-03
chr2:43772121				(0.50, 0.94)							(0.77, 0.97)		(0.79, 1.10)		(0.69, 0.92)	
rs13002812	A/G	0.24	0.29	1.38	2.86E-02	G/A	0.22	0.21	0.24	0.32	0.89	4.34E-02	0.89	1.39E-01	0.89	1.10E-01
chr2:43446616				(1.03, 1.85)							(0.79, 1.00)		(0.75, 1.04)		(0.77, 1.02)	
rs6544660	T/C	0.23	0.29	0.72	3.25E-02	T/C	0.25	0.26	0.23	0.29	0.84	2.78E-03	0.89	1.68E-01	0.77	5.21E-04
chr2:43688496				(0.54, 0.97)							(0.74, 0.94)		(0.76, 1.05)		(0.67, 0.89)	
rs12478601	T/C	0.23	0.29	0.74	4.51E-02	T/C	0.25	0.25	0.23	0.29	0.85	6.89E-03	0.91	2.72E-01	0.78	7.47E-04
chr2:43721508				(0.55, 0.99)							(0.76, 0.96)		(0.77, 1.08)		(0.67, 0.90)	

SNP, single nucleotide polymorphism; Alt, alternate allele; Ref, reference allele; MAF, minor allele frequency; NSCL/P, non-syndromic cleft lip with or without cleft palate; NSCLP, non-syndromic cleft lip with cleft palate; NSCLO, non-syndromic cleft lip only; OR, odds ratio; 95%CI, 95% confidence level.

a In the discovery phase, association analysis was performed according to Alt allele.

b In the replication phase, association analysis was performed according to minor allele.

### *ZFP36L2* Might be the Susceptibility Gene for NSCLO Within 2p21 Locus

Three rare variants of *THADA* and four rare variants of *ZFP36L2* were enrolled in the burden analysis, details about these variants were shown in the Supplementary Table S4. The collective results indicated that rare variants within *ZFP36L2* at 2p21 were significantly associated with increased risk for NSCL/P (*p* = .003, OR = 17.10, 95% CI: 1.99–146.98), NSCLP (*p* = .046, OR = 13.5, 95% CI: 1.22–150.38), and NSCLO (*p* = .0075, OR = 20.73, 95% CI: 2.14–200.53) (Table 2). Interestingly, compared with NSCL/P and NSCLP, *ZFP36L2* conveyed the highest risk to NSCLO with a lower *p* value and larger OR value in our study. However, rare variants within the *THADA* gene did not show a statistically significant association with NSCL/P or NSCLP.

**TABLE 2:** Burden analysis of rare variants in 2p21 locus.

	Gene	Cleft type	Case		Control		*p*-value	OR (95%CI)
			Alt	Ref	Alt	Ref		
Discovery phase	*ZFP36L2*	NSCL/P	5	313	1	1,071	.0030	17.10 (1.99–146.98)
		NSCLP	2	158	1	1,071	.0460	13.50 (1.22–150.38)
		NSCLO	3	155	1	1,071	.0075	20.73 (2.14–200.53)
	*THADA*	NSCL/P	3	315	19	1,065	.4400	0.53 (0.16–1.82)
		NSCLP	3	157	19	1,065	.7600	1.07 (0.31–3.66)
Replication phase	*ZFP36L2*	NSCLO	18	186	7	174	.0489	2.41 (0.98–5.90)

NSCL/P, non-syndromic cleft lip with or without cleft palate (NSCLO and NSCLP); NSCLP, non-syndromic cleft lip with cleft palate; NSCLO, non-syndromic cleft lip only; Alt, alternate allele; Ref, reference allele; OR, odds ratio; 95%CI: 95% confidence interval.

Replication of the burden analysis was conducted by sequencing *ZFP36L2* exons among 204 patients with NSCLO and 181 normal controls. The rare variants were filtered using the same criterion enrolled into the burden analysis, and the result indicated that *ZFP36L2* was also associated with NSCLO (*p* = .0489, OR = 2.41, 95% CI: 0.98–5.90). Based on these findings, we further verified that the 2p21 locus was a risk factor for NSCLO and that the *ZFP36L2* gene in 2p21 is a susceptibility gene for NSCLO in the Han Chinese population.

### *ZFP36L2* Influences Biological Processes in the Etiology of NSCLO

To investigate the potential roles of *ZFP36L2* in the etiology of NSCLO, we performed RNA sequencing on human oral epithelial cell line (GMSM-K) with or without *ZFP36L2* knockdown. Two biological replicates were included for each group.

**FIGURE 2:**
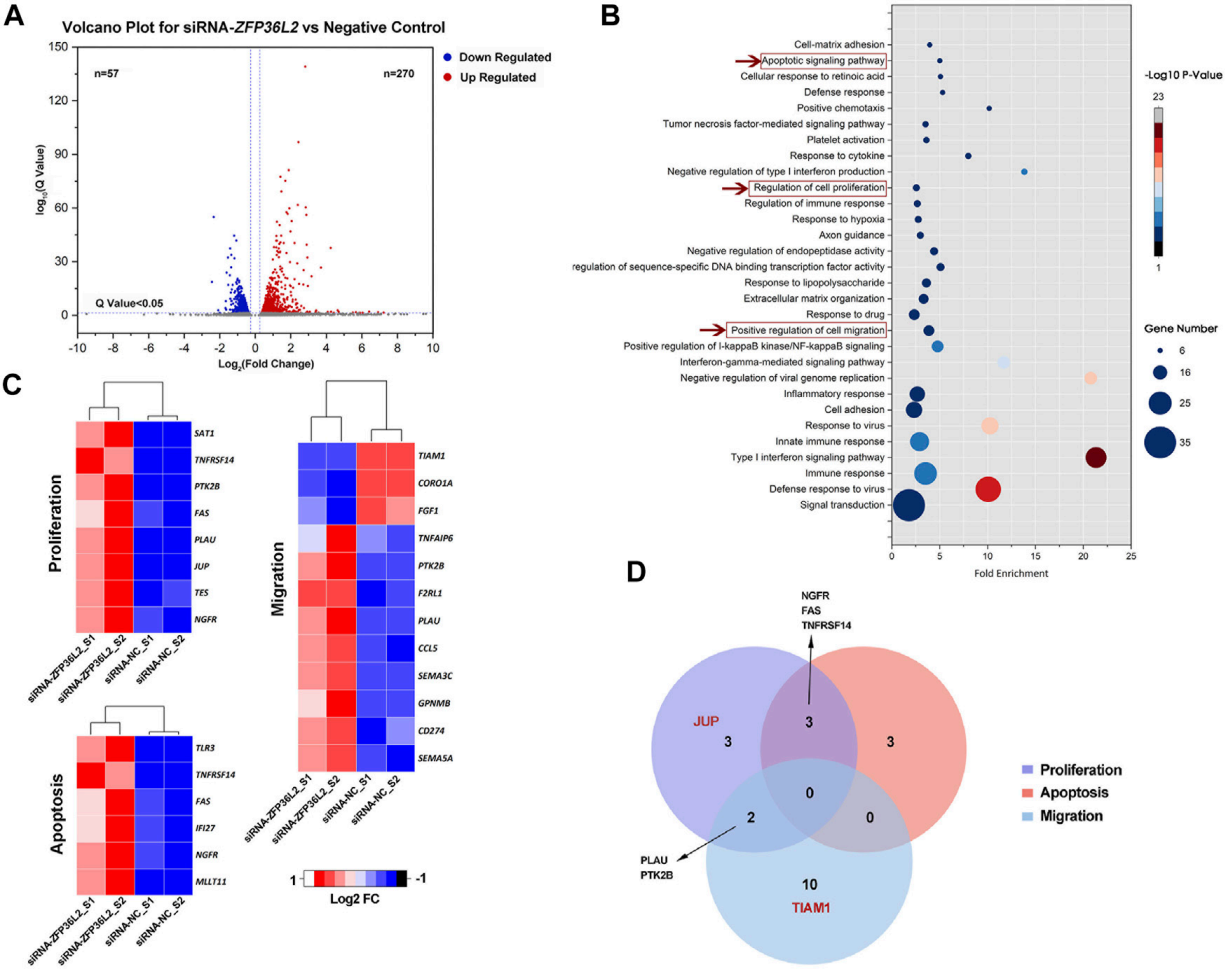
**(A)** Volcano plot for differential gene expression. **(B)** GO biological process terms enriched in DEGs. A total of 67 GO terms were significantly enriched (*p* < .05), the figure shows the top 30 GO terms with the most genes. **(C)** Fold change (Log2 FC) of genes among “Apoptotic signaling pathway,” “regulation of cell proliferation” and “positive regulation of cell migration” biological processes. **(D)** Venn diagram showing number of genes associated with the terms “apoptotic signaling pathway,” “regulation of cell proliferation” and “positive regulation of cell migration.” The red font is indicative of genes where the SNPs associated with NSCLO or NSCPO were located.

Differential gene expression analysis identified 327 differentially expressed genes (DEGs) in total, among which 270 genes were upregulated and 57 genes were downregulated when *ZFP36L2* was knocked down (Figure 2A). GO analysis showed that a series of biological processes were enriched, including terms of “Apoptotic signaling pathway,” “Regulation of cell proliferation” and “Positive regulation of cell migration,” which were associated with lip development (Jiang et al., 2006) (Figure 2B); genes within the two former terms relating to apoptotic and proliferation were totally upregulated, whereas genes within term of “Positive regulation of cell migration” were only partially upregulated (Figure 2C). In addition, we observed that genes involved with the term “regulation of cell proliferation” overlapped with genes involved with the terms “apoptotic signaling pathway” or “positive regulation of cell migration” (Figure 2D).

On the other hand, it is known that the development of the upper lip in mice begins at E10.5, where the medial and lateral nasal processes fuse; later, the maxillary and medial nasal processes grow rapidly and fuse together, marking the completion of lip development (Jiang et al., 2006). To further explore the role of *ZFP36L2* in NSCLO etiology, we searched the literature and found that the *Zfp36l2* gene is expressed in both the maxillary and lateral nasal processes in E10.5 mouse embryos (Supplementary Table S5) (Brunskill et al., 2014). When referring to our previous RNA-seq data of six Chinese Han patients with NSCLO, we noticed that, in the lip tissue, *ZFP36L2* was expressed even higher than *IRF6*, a well-known susceptibility gene for NSCL/P (*ZFP36L2-*Average FPKM: 227.9; *IRF6-*Average FPKM:147.5) (Supplementary Figure S4).

### *ZFP36L2* Target Gene is Associated With NSCLO

**FIGURE 3:**
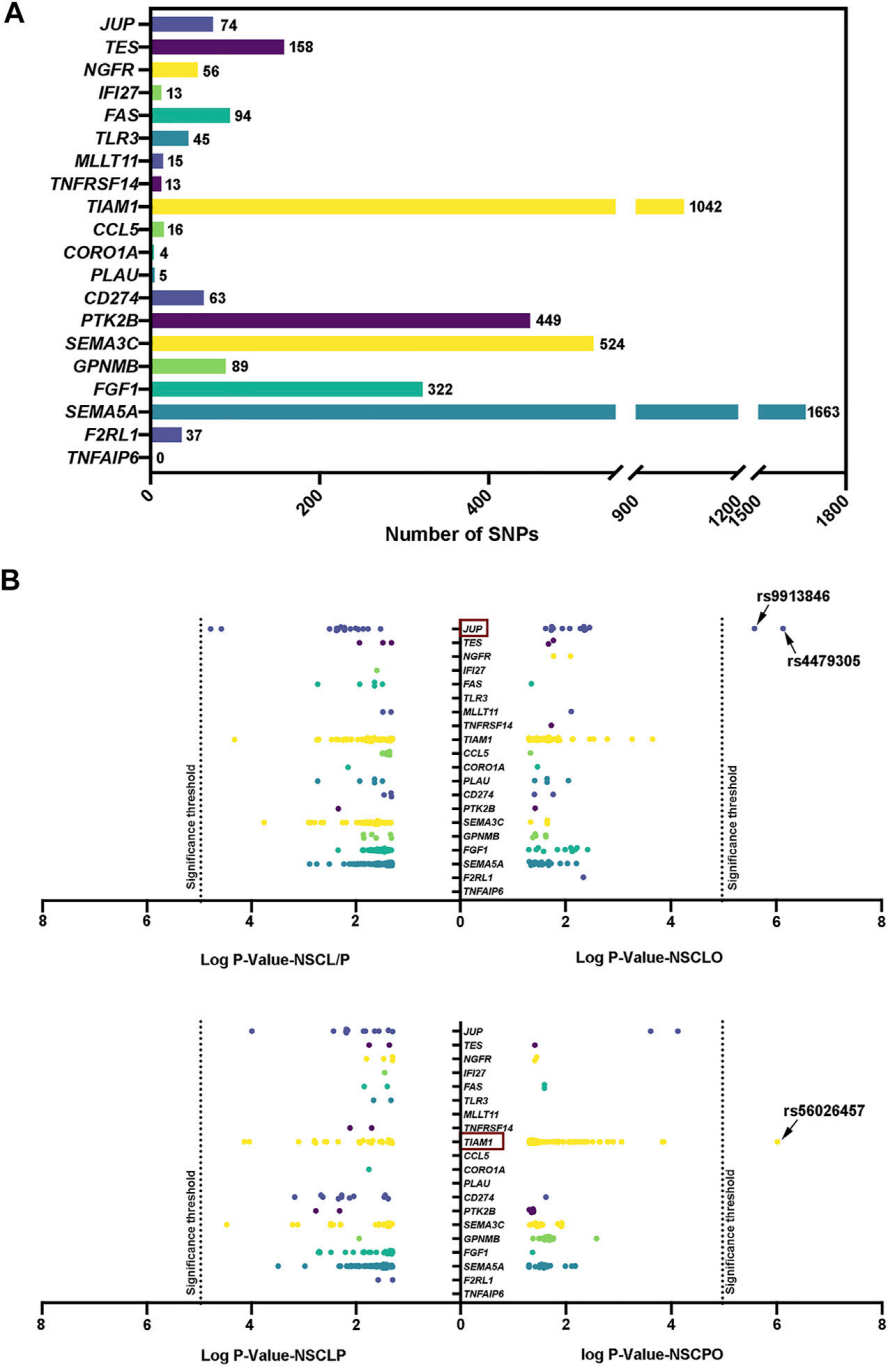
**(A)** Number of SNP within genes associated with the terms “apoptotic signaling pathway,” “regulation of cell proliferation,” and “positive regulation of cell migration; ” **(B)** Recruited SNPs with *p* value pass .05. Log *p* value with base 10 are shown in the figure. The 5.42E-05 significance threshold in the replication phase was adjusted by multiple correction.

Next, from two GWAS databases (Sun et al., 2015; Huang et al., 2019), we retrieved the genotyping data of SNPs at 19 genes within the terms “regulation of cell proliferation” (*NGFR, TES, JUP, PLAU, FAS, PTK2B, TNFRSF14, SAT1*), “apoptotic signaling pathway” (*MLLT11, NGFR, IFI27, FAS, TNFRSF14, TLR3*) and “positive regulation of cell migration” (*SEMA5A, FGF1, CD274, CORO1A, GPNMB, SEMA3C, CCL5, PLAU, TIAM1, F2RL1, PTK2B, TNFAIP6*) that were influenced by *ZFP36L2*. A total of 4,682 SNPs were recruited; we obtained the most SNPs in the *SEMA5A* gene, followed by *TIAM1, SEMA3C, PTK2B,* and *FGF1* genes (Figure 3A). Then, we performed association analysis on these SNPs. When compared to the stringent significance threshold of 1.07E-05 derived from multiple corrections (*p* = .05/4,682), two SNPs within the *JUP* gene were significantly associated with NSCLO: rs4479305 (*p* = 7.42E-07, OR = 0.72, 95% CI: 0.63–0.82) and rs9913846 (*p* = 2.59E-06, OR = 0.73, 95% CI: 0.64–0.83) (Supplementary Table S6). Meanwhile, rs56026457 within the *TIAM1* gene was significantly associated with NSCPO (*p* = 9.68E-07, OR = 0.70, 95% CI: 0.60–0.80), and none were identified to be associated with NSCL/P or NSCLP (Figure 3B).

Furthermore, we noticed that *Jup* was expressed in the medial nasal processes in E10.5 mouse embryos (Supplementary Table S5), whereas *Tiam1* was not. In lip tissues from individuals with NSCLO, *JUP* showed a significantly higher expression level than *TIAM1* (Supplementary Figure S4)*.* These results indicated that *ZFP36L2* might also influence genes participate in lip development.

### *ZFP36L2* Gene Inhibits Cell Proliferation and Induces G2 Phase Arrest

**FIGURE 4:**
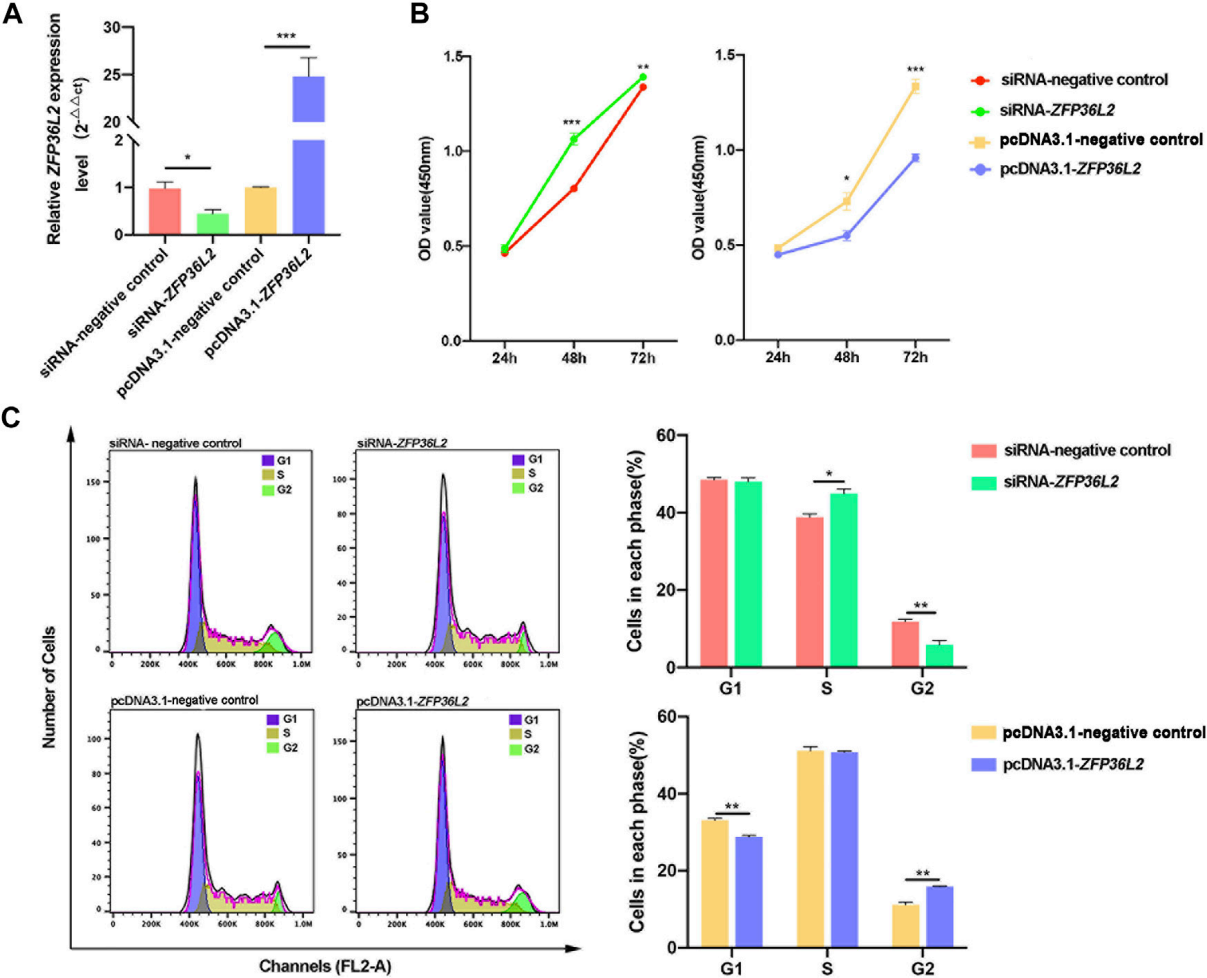
**(A)** RT-qPCR analysis of the expression level of *ZFP36L2* after knockdown or overexpression in the GMSM-K cell line. **(B)** Cell proliferation after knockdown or overexpression of *ZFP36L2* in the GMSM-K cell line. **(C)** Percentage of cells in each cell cycle after knockdown or overexpression of *ZFP36L2* in the GMSM-K cell line. Data is shown as the mean ± SEM. *p < .05; **p < .01; ***p < .001.

Since the *JUP* gene is specifically related to the term “regulation of cell proliferation” (Figure 2D), we investigated proliferation after knockdown or overexpression of *ZFP36L2* in the GMSM-K cell line. RT-qPCR results indicated that the transfection of pcDNA3.1-*ZFP36L2* plasmid and siRNA-*ZFP36L2* could effectively overexpress or inhibit the expression level of *ZFP36L2* gene (Figure 4A). Proliferation assay showed that knockdown of *ZFP36L2* obviously facilitated cell proliferation; however, cell proliferation was significantly inhibited following *ZFP36L2* overexpression (Figure 4B). To clarify this further, cell cycle was also investigated. When *ZFP36L2* was knocked down, cells in G2 phase decreased, accompanied by a significantly increased number of cells in S phase. Conversely, we observed a significant G2 phase arrest accompanied by a reduced number of cells in the G1 phase in *ZFP36L2* overexpressing cells (Figure 4C). These findings indicated that the *ZFP36L2* gene could lead to abnormal lip development by affecting both cell proliferation and the cell cycle.

## Discussion

It is known that NSCL/P has a far more complex genetic architecture than we originally thought, with a variety of genetic risk factors and environmental exposures contributing (Dixon et al., 2011; Boyle et al., 2017). With the advent of the GWAS era, more and more susceptibility genes/loci for NSCL/P have been identified (Birnbaum et al., 2009; Beaty et al., 2010; Mangold et al., 2010; Ludwig et al., 2012; Sun et al., 2015), however, GWASs have only explained a small portion of phenotypic variance since GWAS’ rationale is based on the hypothesis “common disease, common variants,” (Manolio et al., 2009) which results in their lack of ability to detect rare variants. Targeted sequencing, which can detect both common and rare variants, greatly solves the above problems of GWAS. At the same time, findings from GWAS provide a reasonable hypothesis for the targeted region. Therefore, in the present study, we selected the target region of the haplotype around rs7590268 for sequencing in the hope of gaining novel insights into the 2p21 locus. Notably, our results indicated that *ZFP36L2* in 2p21 locus is a susceptibility gene for NSCLO, whereas the previous study only identified *ZFP36L2* and *SIX2* in 2p21 locus to be associated with NSCL/P and NSCPO, respectively (Lin-Shiao et al., 2019; Sweat et al., 2020).

Our evidence can be summarized into two parts, the first being bioinformatics-related. Association analysis based on a large cohort and stringent significance threshold showed that most SNPs in 2p21 locus were associated with NSCLO, from which we inferred that 2p21 was a susceptibility locus for NSCLO. We annotated the six statistically significant SNPs using HaploReg v4.1 (https://pubs.broadinstitute.org/mammals/haploreg/haploreg.php) (Supplementary Table S7), finding that different alleles of these six SNPs resulted in altered motifs, except for rs12990267. Among these, the T allele of rs201795193 altered most motifs, including Pax7, which is critical for myogenic satellite cell specification (Seale et al., 2000). The T allele of rs74343467 altered the motif of E2F, a family of transcription factors important for life and death due to their involvement in various biological processes such as DNA replication, cell differentiation and proliferation (Helin, 1998; Polager and Ginsberg, 2008). Furthermore, rs6544660 and rs12478601 were shown to be present in regulatory elements among epidermal keratinocyte primary cells and influenced *ZFP36L2* expression levels in whole blood and naive monocytes, although all six SNPs were in the intronic region of the *THADA* gene. These results suggested that these SNPs might be functional. Burden analysis specified our inference, that is, the *ZFP36L2* gene in 2p21 locus was more significantly associated with NSCLO. *ZFP36L2* conferred the highest OR of 20.73, and this association was also statistically significant in subsequent validation (*p* = .0489, OR = 2.41, 95% CI: .98–5.90).

Functional analysis of the *ZFP36L2* was carried out to further determine its association with NSCLO. We found that *Zfp36l2* gene was expressed in the facial processes related to mouse lip development (Supplementary Table S5). In human lip tissue, we also observed that *ZFP36L2* gene expression level was even higher than *IRF6*, for which the etiology of NSCLO was quite clear since a relatively high expression level of *IRF6* would lead to the occurrence of NSCLO (Huang et al., 2019). Additionally, through RNA-seq, we observed that *ZFP36L2* affected three biological processes related to proliferation, migration, and apoptosis, which are critical in lip development (Jiang et al., 2006). In this section, we noticed that DEGs appeared to be most enriched in immune-related biological processes, this may be due to the involvement of *ZFP36L2* in thymic development and T lymphoblastic leukemia (Hodson et al., 2010). Lastly, we conducted an association analysis of the SNPs within genes involved in these three biological processes and NSOFC, finding that two of the three statistically significant SNPs were specifically associated with NSCLO. Considering all the findings, we rationally proposed that *ZFP36L2* in the 2p21 locus is a susceptibility gene for NSCLO.

We attempted to address how the *ZFP36L2* gene leads to NSCLO. We noticed that four genes (*Ngfr, Tes, Jup,* and *Sat1*) associated with the term “regulation of cell proliferation” were expressed in the medial nasal, lateral nasal or maxillary processes. However, only two genes (*Mllt11* and *Ngfr* genes) associated with the term “apoptotic signaling pathway” and three genes (*Sema5a*, *Coro1a* and *Tnfaip6* gene) associated with the term “positive regulation of cell migration” were expressed in the three upper lip-related processes (Supplementary Table S5) (Brunskill et al., 2014). Furthermore, the two SNPs associated with NSCLO were both occurring within the proliferation-related gene *JUP*, which was influenced by *ZFP36L2* (Figure 2D and Figure 3B). Therefore, we speculated that *ZFP36L2* might lead to NSCLO by influencing cell proliferation, and our study proved that *ZFP36L2* negatively regulates cell proliferation and induces cell arrest in the G2 phase of GMSM-K. Our results were consistent with those of previous studies, that is, *ZFP36L2* inhibited cell proliferation through a cyclin D-dependent and p53-independent pathway (J. Liu et al., 2018; Suk et al., 2018). As for the effect of *ZFP36L2* on the cell cycle, the results are slightly varied. Fat-Moon Suk et al. observed that *ZFP36L2* led to G1 phase arrest and thus resulted in a decreased S phase (Suk et al., 2018), whereas several other studies have shown that *ZFP36L2* might be a key protein in S phase progression control in the case of genome instability (Galloway et al., 2016; Vogel et al., 2016; Noguchi et al., 2018). In our study, we noticed that as long as the cells in G2 phase were influenced, the subsequent G1 or S phases would be affected more or less. Thus, it is certain that *ZFP36L2* affects the cell cycle, but its effects may be slightly modified in different cell types.

In summary, our study comprehensively illustrated the important role of the *ZFP36L*2 gene in the etiology of NSCLO and proposed that *ZFP36L2* is a novel susceptibility gene for NSCLO among the Han Chinese population. Further research is required to address limitations of this study. All samples recruited in our study were from the Han Chinese population; due to the genetic heterogeneity among different populations, further verification should be conducted in other populations. Additionally, to clarify the role of *ZFP36L2* in NSCLO intuitively, follow-up experiments in animal models are necessary.

## Data Availability

All the datasets generated in this article were shown in the main text and the Supplementary Material. Any questions about the data, please contact to zhonglinjia@sina.com.
