# Supplementary material for: Targeted Re-Sequencing of the 2p21 Locus Identifies Non-Syndromic Cleft Lip Only Novel Susceptibility Gene ZFP36L2
Source: Front Genet. 2022 Feb 9;13:802229. doi: 10.3389/fgene.2022.802229 (PMC8886408; doi:10.3389/fgene.2022.802229)
Supplement: Supplementary file 1 [file DataSheet1.docx]

**Targeted re-sequencing of the 2p21 locus identifies non-syndromic cleft lip only novel susceptibility gene *ZFP36L2***

Mujia Li ^1,#^, Jia-Yu Shi^2,#^, Qiu-Shuang Zhu^1^, Bing Shi^1,^ , Zhong-Lin Jia^1,*^


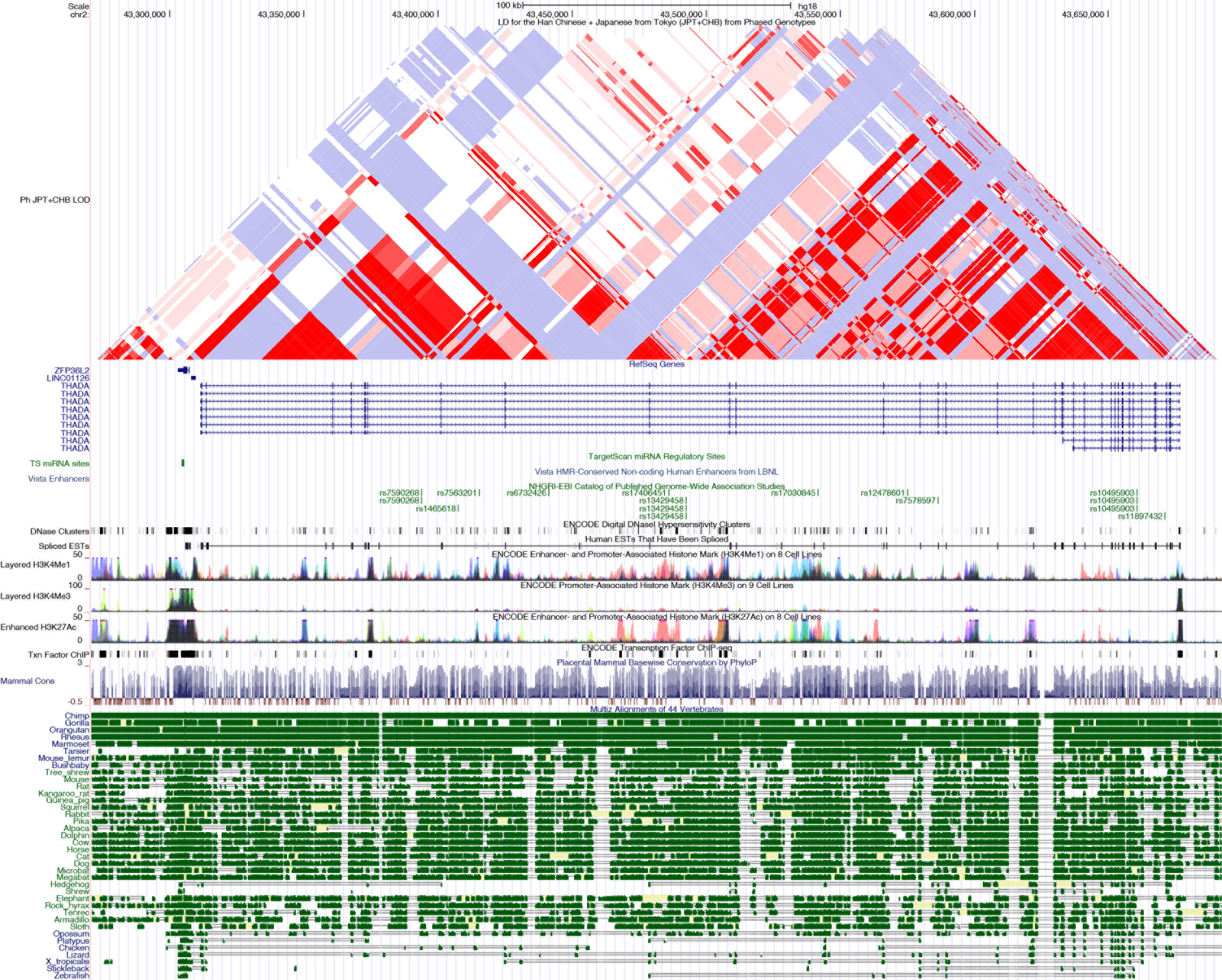


**Supplementary Figure 1** Targeted sequencing region at 2p21 locus，including exons, introns, and the inter-gene region.


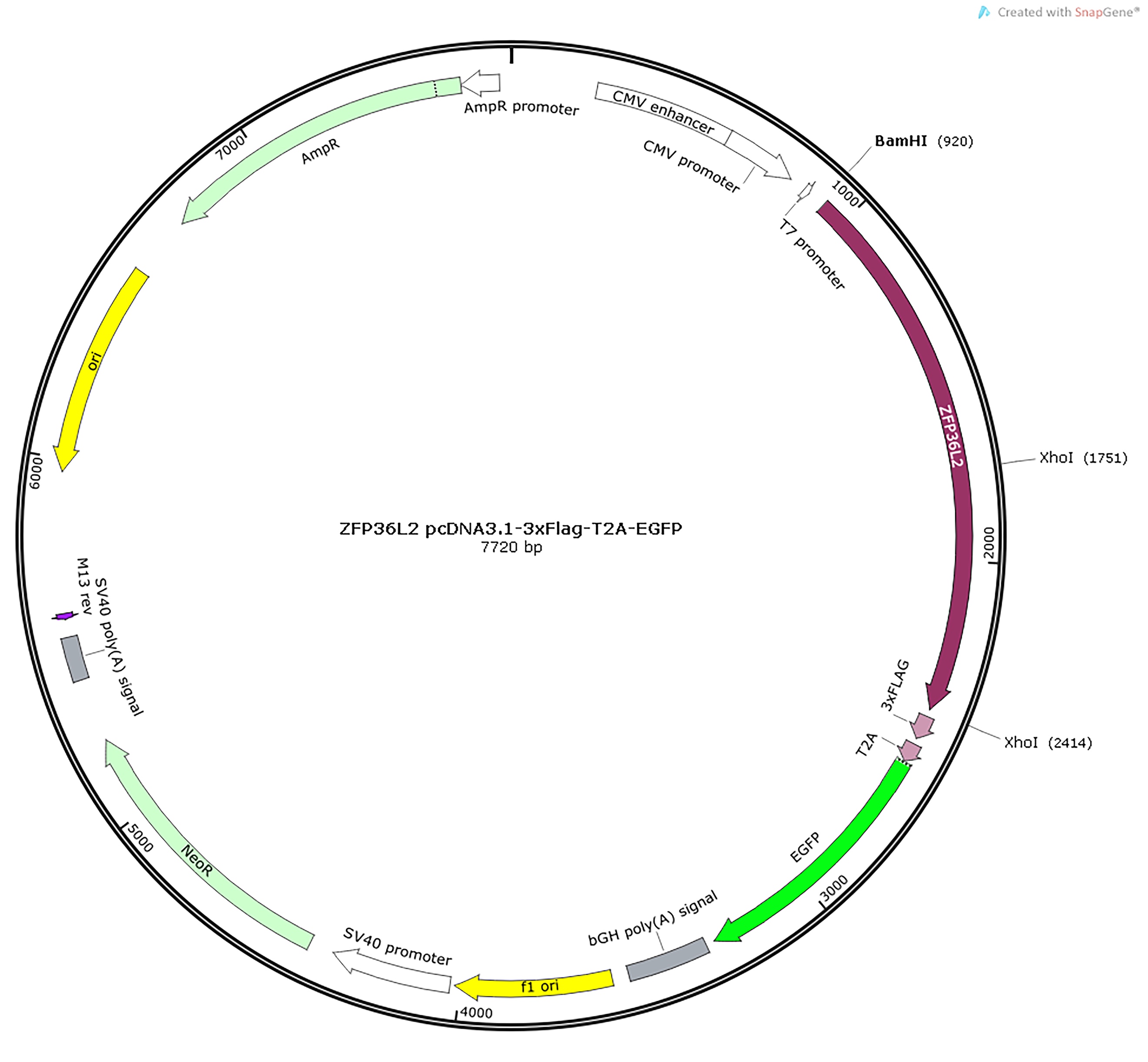


**Supplementary Figure 2** Structure of the recombinant pcDNA3.1- *ZFP36L2*


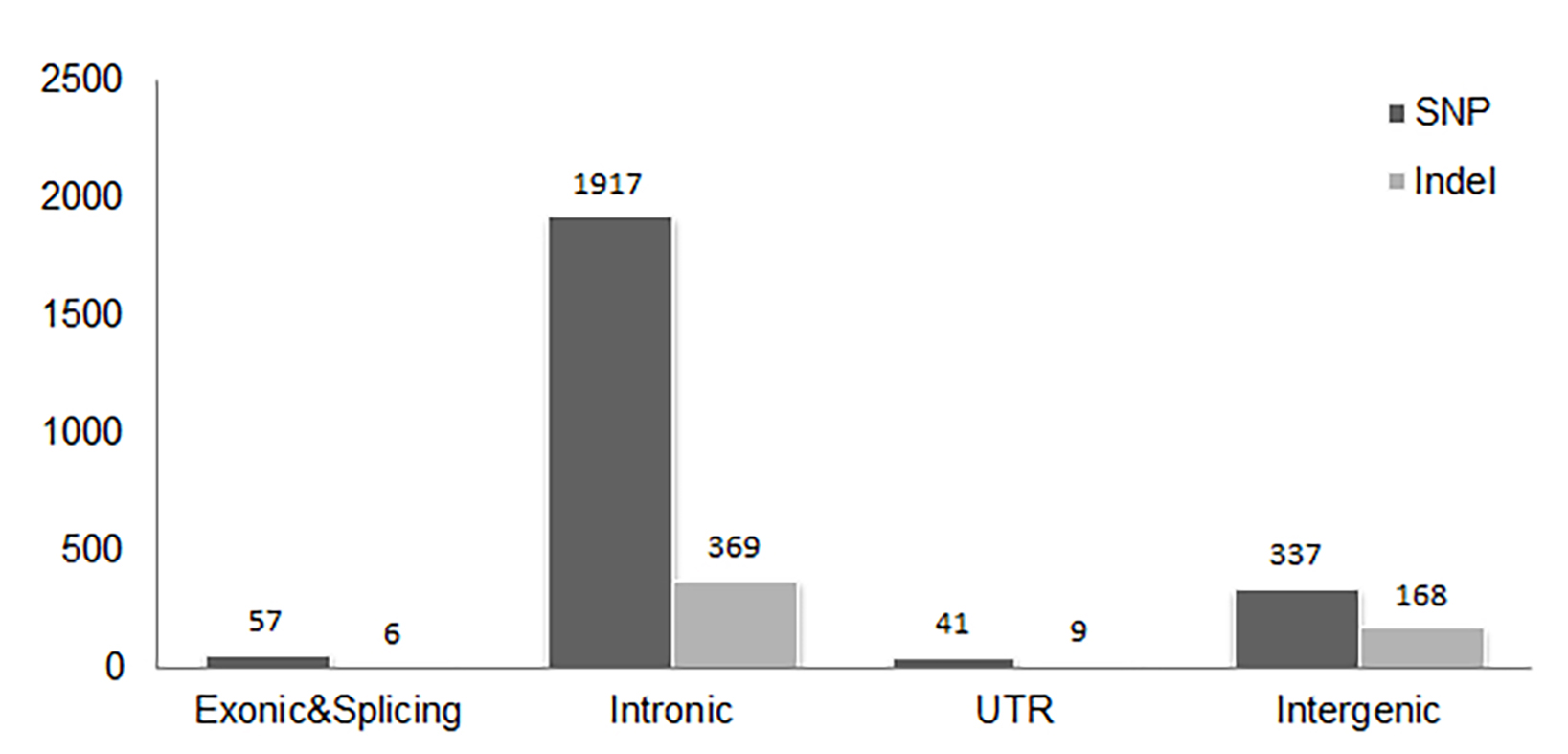


**Supplementary Figure 3** Summary of the variations.

**Supplementary Figure 4** Expression level of *ZFP36L2*, *IRF6, JUP* and *TIAM1* gene from lip tissue of six NSCLO patients. FPKM, Fragments Per Kilobase Million. *, P<0.05, ****, P<0.0001

| **Supplementary Table 1** Samples recruited in the study | | | | | |
| --- | --- | --- | --- | --- | --- |
|  |  | NSCL/P | NSCLP | NSCLO | Control |
| Discovery Phase | Association analysis | 159 | 80 | 79 | 542 |
|  | Burden analysis |  |  |  |  |
| Replication Phase | Association analysis | 1626 | 579 | 1047 | 2255 |
|  | Burden analysis | / | / | 204 | 181 |
| **Note：**NSCL/P, non-syndromic cleft lip with or without cleft palate; NSCLP, non-syndromic cleft lip with cleft palate; NSCLO, non-syndromic cleft lip only. | | | | | |
|  | | | | | |

| **Supplementary Table 2** The sequence of RT-qPCR primers | |
| --- | --- |
| Gene | Primer Sequence (5'-3') |
| *ZFP36L2* | F: TCCAGAAACATGTCGACCAC |
|  | R: CCAGCATGTTGTTCAGGTTG |
| *GAPDH* | F: TGACTTCAACAGCGACACCCA |
|  | R: CACCCTGTTGCTGTAGCCAAA |

| **Supplementary Table 3** Hardy-Weinberg test for the SNPs in 2p21 locus | | | | | | | | | | | |
| --- | --- | --- | --- | --- | --- | --- | --- | --- | --- | --- | --- |
| SNP | Position(hg19) | A1 | Discovery phase | | | |  | Replication phase | | | |
|  |  |  | GENO | O(HET) | E(HET) | P |  | GENO | O(HET) | E(HET) | P |
| rs201795193 | chr2:43771991 | T | 15/143/381 | 0.27 | 0.27 | 0.7486 |  | 96/651/1046 | 0.36 | 0.36 | 0.7426 |
| rs12990267 | chr2:43770432 | G | 0/42/496 | 0.08 | 0.08 | 1.0000 |  | 103/670/1020 | 0.37 | 0.37 | 0.6544 |
| rs199721109 | chr2:43772391 | G | 22/199/321 | 0.37 | 0.35 | 0.2184 |  | 103/670/1020 | 0.37 | 0.37 | 0.6544 |
| rs74343467 | chr2:43772121 | T | 19/235/287 | 0.43 | 0.38 | 0.0004 |  | 134/752/907 | 0.42 | 0.41 | 0.2229 |
| rs13002812 | chr2:43446616 | G | 45/234/263 | 0.43 | 0.42 | 0.5389 |  | 176/782/835 | 0.44 | 0.43 | 0.7433 |
| rs6544660 | chr2:43688496 | T | 37/242/263 | 0.45 | 0.41 | 0.0766 |  | 140/746/907 | 0.42 | 0.41 | 0.4527 |
| rs12478601 | chr2:43721508 | T | 38/234/266 | 0.43 | 0.41 | 0.1734 |  | 136/753/904 | 0.42 | 0.41 | 0.2472 |
| **Note:** SNP, Single Nucleotide Polymorphism; A1, minor allele; GENO,Genotype counts; O(HET), Observed heterozygosity; E(HET), Expected heterozygosity; P, Hardy-Weinberg test P value. | | | | | | | | | | | |

| **Supplementary Table 4** Variants enrolled in the burden analysis | | | | | | | | |
| --- | --- | --- | --- | --- | --- | --- | --- | --- |
| Gene | Variant | Position(hg19) | Phenotype | Variant type | Function prediction | | | |
|  |  |  |  |  | SIFT | Polyphen2_  HDIV | Mutation  Taster | CADD |
| *ZFP36L2* | NM_006887.4:exon2:c.C406T:p.H136Y | chr2:43452537 | NSCLP | Missense | 0.017, D | 0.699, P | Disease causing | 12.48 |
|  | NM_006887.4:exon2:c.C236A:p.P79Q | chr2:43452707 | NSCLP, NSCLO | Missense | 0.017, D | 0.019, B | Polymorphism | 16.22 |
|  | NM_006887.4:exon2:c.C199T:p.P67S | chr2:43452744 | NSCLO | Missense | 0.031, D | 0.066, B | Disease causing | 16.34 |
|  | NM_006887.4:exon2:c.T89C:p.M30T | chr2:43452854 | NSCLO | Missense | 0.012, D | 0.982, D | Disease causing | 22.4 |
| *THADA* | NM_001083953.1:exon13:c.G1909T:p.V637L | chr2:43799001 | NSCLP | Missense | 0.105, T | 1.0, D | Disease causing | 24.3 |
|  | NM_001083953.1:exon12:c.C1828T:p.L610F | chr2:43800033 | NSCLP | Missense | 0.006, D | 1.0, D | Disease causing | 29.2 |
|  | NM_001083953.1:exon11:c.A1478T:p.Y493F | chr2:43801726 | NSCLP | Missense | 0.004, D | 1.0, D | Disease causing | 26.1 |
| Note: NSCLP, non-syndromic cleft lip with cleft palate; NSCLO, non-syndromic cleft lip only. The recruitment threshold for rare variants: SIFT scores <0.05; Polyphen2_HDIV scores ≥0.957; Mutation Taster, "Disease causing"; CADD scores >10. D, damaging; T, tolerated; B, benign; P, possibly damaging. | | | | | | | | |

| **Supplementary Table 5** FPKM of genes from E10.5 Mouse Embryos | | | | | |  |
| --- | --- | --- | --- | --- | --- | --- |
|  | **Gene Symbol** | **E10.5 Mouse Embryo** | | | |  |
|  |  | **Lateral_Nasal_Prominence** | **Medial_Nasal_Prominence** | **MaxA_Prominence** |  |  |
|  | ***Tlr3*** |  |  |  | **Apoptosis** |  |
|  | ***Ifi27*** |  |  |  |  |  |
|  | ***Mllt11*** | 0.5179 | **5.0462** | 0.3189 |  |  |
| **Proliferation** | ***Ngfr*** | **1.8871** | 0.8876 | **2.2943** |  |  |
|  | ***Tnfrsf14*** |  |  |  |  |  |
|  | ***Fas*** |  |  |  |  |  |
|  | ***Tes*** | **1.1991** | 0.2514 | **1.9255** |  |  |
|  | ***Jup*** | 0.9631 | **1.9326** | 0.6587 |  |  |
|  | ***Sat1*** | **1.4462** | **1.0898** | 0.5860 |  |  |
|  | ***Plau*** |  |  |  | **Migration** |  |
|  | ***Ptk2b*** |  |  |  |  |  |
|  | ***Sema5a*** | 0.9784 | 0.4570 | **1.7604** |  |  |
|  | ***Coro1a*** | 0.9291 | **1.2889** | **1.4406** |  |  |
|  | ***Tnfaip6*** | **3.6966** | **3.6966** | **3.6966** |  |  |
|  | ***Fgf1*** |  |  |  |  |  |
|  | ***Cd274*** |  |  |  |  |  |
|  | ***Gpnmb*** |  |  |  |  |  |
|  | ***Sema3c*** |  |  |  |  |  |
|  | ***Ccl5*** |  |  |  |  |  |
|  | ***Tiam1*** |  |  |  |  |  |
|  | ***F2rl1*** |  |  |  |  |  |
|  | ***Zfp36l2*** | **2.3103** | 0.8371 | **1.3832** |  |  |
|  | ***Thada*** | 0.8928 | 0.9785 | 0.6988 |  |  |
| **Note:** FPKM, Fragments Per Kilobase per Million. The RNA-seq FPKM table was normalized using a baseline of 1 FPKM (a level of gene expression unlikely to be significant in these assays) followed by global median normalization to give a table of ratio values. | | | | | |  |
|  |  |  |  |  |  |  |
|  |  |  |  |  |  |  |

| **Supplementary Table 6** Recruited SNPs within *ZFP36L2* target biological processes with P value pass 0.05 among NSCLO | | | | | | | | | | | | | | | | | |
| --- | --- | --- | --- | --- | --- | --- | --- | --- | --- | --- | --- | --- | --- | --- | --- | --- | --- |
| CHR | SNP | Position(hg19) | A1 | MAF | |  | HWE | | | |  | | OR | 95%CI | | P |  |
|  |  |  |  | NSCLO | Control |  | GENO | O(HET) | E(HET) | P_HWE_ |  | |  | L95 | U95 |  |  |
| 17 | rs4479305 | 39937704 | A | 0.39 | 0.50 |  | 450/884/459 | 0.49 | 0.50 | 0.5708 |  | | 0.72 | 0.63 | 0.82 | 7.42E-07 |  |
| 17 | rs9913846 | 39937376 | C | 0.58 | 0.49 |  | 465/896/432 | 0.50 | 0.50 | 1.0000 |  | | 0.73 | 0.64 | 0.83 | 2.59E-06 |  |
| 21 | rs56026457 | 32543379 | C | 0.33 | 0.46 |  | 383/878/532 | 0.49 | 0.50 | 0.5682 | |  | 0.78 | 0.69 | 0.89 | 2.22E-04 |  |
| 21 | rs141465101 | 32579093 | C | 0.03 | 0.02 |  | 1/84/1708 | 0.05 | 0.05 | 1.0000 |  | | 2.02 | 1.36 | 3.02 | 5.44E-04 |  |
| 21 | rs561535 | 32775818 | T | 0.11 | 0.33 |  | 209/771/813 | 0.43 | 0.44 | 0.2015 |  | | 0.81 | 0.71 | 0.93 | 2.91E-03 |  |
| 21 | rs187687052 | 32871233 | C | 0.03 | 0.03 |  | 1/89/1703 | 0.05 | 0.05 | 1.0000 |  | | 1.69 | 1.19 | 2.41 | 3.47E-03 |  |
| 17 | rs35759479 | 39935255 | G | 0.47 | 0.44 |  | 348/881/564 | 0.49 | 0.49 | 0.9236 |  | | 1.22 | 1.07 | 1.39 | 3.48E-03 |  |
| 5 | rs10070885 | 142020380 | T | 0.16 | 0.15 |  | 40/469/1284 | 0.26 | 0.26 | 0.7848 |  | | 1.30 | 1.09 | 1.54 | 3.80E-03 |  |
| 17 | rs4796607 | 39934989 | C | 0.47 | 0.44 |  | 349/882/562 | 0.49 | 0.49 | 0.9237 |  | | 1.22 | 1.06 | 1.39 | 4.06E-03 |  |
| 17 | rs7211296 | 39934890 | G | 0.47 | 0.44 |  | 351/879/563 | 0.49 | 0.49 | 0.8108 |  | | 1.21 | 1.06 | 1.38 | 4.38E-03 |  |
| 17 | rs12948083 | 39936570 | G | 0.50 | 0.44 |  | 351/882/560 | 0.49 | 0.49 | 0.9237 |  | | 1.21 | 1.06 | 1.38 | 4.46E-03 |  |
| 5 | rs616235 | 76128521 | G | 0.10 | 0.14 |  | 28/443/1322 | 0.25 | 0.24 | 0.2009 |  | | 0.75 | 0.61 | 0.91 | 4.57E-03 |  |
| 17 | rs35114896 | 39935056 | A | 0.47 | 0.44 |  | 344/881/568 | 0.49 | 0.49 | 0.9617 |  | | 1.21 | 1.06 | 1.38 | 5.19E-03 |  |
| 5 | rs6884797 | 142008657 | A | 0.22 | 0.20 |  | 66/592/1135 | 0.33 | 0.32 | 0.3405 |  | | 1.25 | 1.07 | 1.47 | 6.02E-03 |  |
| 5 | rs375093050 | 9262929 | C | 0.02 | 0.03 |  | 3/89/1701 | 0.05 | 0.05 | 0.1259 |  | | 1.72 | 1.17 | 2.54 | 6.17E-03 |  |
| 5 | rs36033246 | 142008944 | C | 0.15 | 0.14 |  | 33/426/1334 | 0.24 | 0.24 | 1.0000 |  | | 1.29 | 1.07 | 1.56 | 6.50E-03 |  |
| 21 | rs76015126 | 32624580 | G | 0.04 | 0.06 |  | 8/185/1600 | 0.10 | 0.11 | 0.2637 |  | | 0.67 | 0.51 | 0.90 | 7.22E-03 |  |
| 21 | rs9647059 | 32527012 | A | 0.07 | 0.10 |  | 26/315/1452 | 0.18 | 0.18 | 0.0709 |  | | 0.74 | 0.60 | 0.92 | 7.30E-03 |  |
| 21 | rs79676037 | 32521280 | C | 0.29 | 0.27 |  | 106/747/940 | 0.42 | 0.39 | 0.0079 |  | | 1.23 | 1.06 | 1.42 | 7.37E-03 |  |
| 5 | rs34115593 | 142010143 | A | 0.15 | 0.14 |  | 33/428/1332 | 0.24 | 0.24 | 0.9209 |  | | 1.29 | 1.07 | 1.55 | 7.45E-03 |  |
| 1 | rs6674232 | 151038920 | C | 0.01 | 0.03 |  | 4/108/1681 | 0.06 | 0.06 | 0.1123 |  | | 0.56 | 0.37 | 0.86 | 7.72E-03 |  |
| 5 | rs71587226 | 142007802 | A | 0.15 | 0.14 |  | 34/418/1341 | 0.23 | 0.23 | 0.8401 |  | | 1.28 | 1.07 | 1.55 | 7.88E-03 |  |
| 17 | rs11466155 | 47588000 | T | 0.04 | 0.10 |  | 12/330/1451 | 0.18 | 0.18 | 0.1830 |  | | 0.73 | 0.58 | 0.92 | 8.03E-03 |  |
| 17 | rs72835674 | 39939970 | A | 0.61 | 0.49 |  | 449/931/413 | 0.52 | 0.50 | 0.1081 |  | | 1.20 | 1.05 | 1.37 | 8.27E-03 |  |
| 10 | rs2227564 | 75673101 | T | 0.36 | 0.39 |  | 286/823/684 | 0.46 | 0.48 | 0.1497 |  | | 1.19 | 1.05 | 1.35 | 8.75E-03 |  |
| 5 | rs17217037 | 142015345 | G | 0.16 | 0.15 |  | 39/453/1301 | 0.25 | 0.25 | 1.0000 |  | | 1.26 | 1.06 | 1.50 | 1.02E-02 |  |
| 5 | rs146126615 | 9521252 | G | 0.03 | 0.01 |  | 0/53/1740 | 0.03 | 0.03 | 1.0000 |  | | 1.86 | 1.14 | 3.03 | 1.26E-02 |  |
| 21 | rs2300343 | 32603442 | A | 0.07 | 0.09 |  | 14/293/1486 | 0.16 | 0.16 | 1.0000 |  | | 0.74 | 0.59 | 0.94 | 1.28E-02 |  |
| 21 | rs1977905 | 32668006 | T | 0.02 | 0.01 |  | 0/38/1755 | 0.02 | 0.02 | 1.0000 |  | | 2.32 | 1.19 | 4.51 | 1.32E-02 |  |
| 21 | rs62222869 | 32895421 | C | 0.08 | 0.10 |  | 13/321/1459 | 0.18 | 0.17 | 0.3465 |  | | 0.75 | 0.60 | 0.94 | 1.34E-02 |  |
| 21 | rs2246922 | 32561775 | A | 0.10 | 0.12 |  | 27/376/1390 | 0.21 | 0.21 | 0.7379 |  | | 0.77 | 0.63 | 0.95 | 1.36E-02 |  |
| 21 | rs2246927 | 32561980 | G | 0.10 | 0.12 |  | 27/376/1390 | 0.21 | 0.21 | 0.7379 |  | | 0.77 | 0.63 | 0.95 | 1.36E-02 |  |
| 21 | rs2284479 | 32562437 | C | 0.10 | 0.12 |  | 27/376/1390 | 0.21 | 0.21 | 0.7379 |  | | 0.77 | 0.63 | 0.95 | 1.36E-02 |  |
| 21 | rs2284543 | 32881688 | A | 0.08 | 0.10 |  | 13/317/1463 | 0.18 | 0.17 | 0.4137 |  | | 0.75 | 0.60 | 0.94 | 1.41E-02 |  |
| 21 | rs8132656 | 32882563 | A | 0.08 | 0.10 |  | 13/317/1463 | 0.18 | 0.17 | 0.4137 |  | | 0.75 | 0.60 | 0.94 | 1.41E-02 |  |
| 21 | rs11702341 | 32892160 | A | 0.08 | 0.10 |  | 13/317/1463 | 0.18 | 0.17 | 0.4137 |  | | 0.75 | 0.60 | 0.94 | 1.41E-02 |  |
| 21 | rs139559444 | 32892240 | A | 0.08 | 0.10 |  | 13/317/1463 | 0.18 | 0.17 | 0.4137 |  | | 0.75 | 0.60 | 0.94 | 1.41E-02 |  |
| 21 | rs3787685 | 32893239 | A | 0.08 | 0.10 |  | 13/317/1463 | 0.18 | 0.17 | 0.4137 |  | | 0.75 | 0.60 | 0.94 | 1.41E-02 |  |
| 5 | rs34702386 | 142017217 | A | 0.15 | 0.14 |  | 31/424/1338 | 0.24 | 0.23 | 0.7628 |  | | 1.26 | 1.05 | 1.52 | 1.42E-02 |  |
| 21 | rs142776261 | 32590687 | T | 0.05 | 0.06 |  | 9/200/1584 | 0.11 | 0.11 | 0.3001 |  | | 0.71 | 0.54 | 0.94 | 1.52E-02 |  |
| 21 | rs117390252 | 32751175 | G | 0.01 | 0.02 |  | 2/73/1718 | 0.04 | 0.04 | 0.1955 |  | | 0.45 | 0.24 | 0.86 | 1.57E-02 |  |
| 9 | rs76681637 | 5460458 | C | 0.08 | 0.06 |  | 6/218/1569 | 0.12 | 0.12 | 0.8428 |  | | 0.71 | 0.53 | 0.94 | 1.71E-02 |  |
| 17 | rs11650969 | 39934077 | G | 0.41 | 0.44 |  | 354/874/565 | 0.49 | 0.49 | 0.6321 |  | | 1.17 | 1.03 | 1.34 | 1.81E-02 |  |
| 21 | rs475142 | 32811608 | C | 0.22 | 0.18 |  | 56/534/1203 | 0.30 | 0.30 | 0.8104 |  | | 1.21 | 1.03 | 1.42 | 1.84E-02 |  |
| 1 | rs180732608 | 2490210 | T | 0.01 | 0.01 |  | 1/50/1742 | 0.03 | 0.03 | 0.3128 |  | | 1.85 | 1.11 | 3.09 | 1.85E-02 |  |
| 5 | rs11954325 | 9088874 | T | 0.03 | 0.05 |  | 4/168/1621 | 0.09 | 0.09 | 1.0000 |  | | 0.68 | 0.49 | 0.94 | 1.86E-02 |  |
| 21 | rs200313183 | 32776809 | G | 0.06 | 0.03 |  | 2/103/1688 | 0.06 | 0.06 | 0.6707 |  | | 1.53 | 1.07 | 2.18 | 1.87E-02 |  |
| 21 | rs11702351 | 32869357 | A | 0.08 | 0.10 |  | 13/317/1463 | 0.18 | 0.17 | 0.4137 |  | | 0.77 | 0.61 | 0.96 | 1.99E-02 |  |
| 21 | rs11702338 | 32869453 | T | 0.08 | 0.10 |  | 13/317/1463 | 0.18 | 0.17 | 0.4137 |  | | 0.77 | 0.61 | 0.96 | 1.99E-02 |  |
| 21 | rs55993777 | 32863882 | C | 0.08 | 0.10 |  | 13/319/1461 | 0.18 | 0.17 | 0.4141 |  | | 0.77 | 0.61 | 0.96 | 2.00E-02 |  |
| 21 | rs67500730 | 32554394 | A | 0.12 | 0.14 |  | 44/426/1323 | 0.24 | 0.25 | 0.1775 |  | | 0.80 | 0.66 | 0.97 | 2.01E-02 |  |
| 21 | rs2236614 | 32559817 | A | 0.10 | 0.12 |  | 28/375/1390 | 0.21 | 0.21 | 0.6546 |  | | 0.78 | 0.64 | 0.96 | 2.03E-02 |  |
| 5 | rs253634 | 9091164 | A | 0.15 | 0.16 |  | 38/480/1275 | 0.27 | 0.26 | 0.4169 |  | | 0.80 | 0.67 | 0.97 | 2.04E-02 |  |
| 21 | rs55662795 | 32519028 | C | 0.37 | 0.36 |  | 232/840/721 | 0.47 | 0.46 | 0.6460 |  | | 1.17 | 1.03 | 1.34 | 2.04E-02 |  |
| 5 | rs253638 | 9094295 | A | 0.15 | 0.16 |  | 38/481/1274 | 0.27 | 0.26 | 0.3693 |  | | 0.80 | 0.67 | 0.97 | 2.08E-02 |  |
| 5 | rs253644 | 9098636 | C | 0.15 | 0.16 |  | 38/481/1274 | 0.27 | 0.26 | 0.3693 |  | | 0.80 | 0.67 | 0.97 | 2.08E-02 |  |
| 21 | rs34403849 | 32869447 | T | 0.08 | 0.10 |  | 13/315/1465 | 0.18 | 0.17 | 0.4912 |  | | 0.77 | 0.61 | 0.96 | 2.08E-02 |  |
| 21 | rs2833305 | 32521396 | T | 0.34 | 0.34 |  | 182/845/766 | 0.47 | 0.45 | 0.0231 |  | | 1.18 | 1.03 | 1.36 | 2.10E-02 |  |
| 7 | rs78788246 | 115881405 | C | 0.01 | 0.02 |  | 1/79/1713 | 0.04 | 0.04 | 0.6032 |  | | 0.54 | 0.32 | 0.91 | 2.11E-02 |  |
| 21 | rs8132120 | 32558712 | A | 0.10 | 0.12 |  | 27/371/1395 | 0.21 | 0.21 | 0.6516 |  | | 0.79 | 0.64 | 0.97 | 2.19E-02 |  |
| 21 | rs73191666 | 32559974 | C | 0.10 | 0.12 |  | 27/371/1395 | 0.21 | 0.21 | 0.6516 |  | | 0.79 | 0.64 | 0.97 | 2.19E-02 |  |
| 7 | rs10247306 | 80385604 | T | 0.26 | 0.23 |  | 90/658/1045 | 0.37 | 0.36 | 0.3227 |  | | 1.20 | 1.03 | 1.40 | 2.20E-02 |  |
| 7 | rs534891159 | 80492985 | G | 0.03 | 0.02 |  | 1/63/1729 | 0.04 | 0.04 | 0.4461 |  | | 1.60 | 1.07 | 2.39 | 2.21E-02 |  |
| 7 | rs573687754 | 80492999 | G | 0.03 | 0.02 |  | 1/63/1729 | 0.04 | 0.04 | 0.4461 |  | | 1.60 | 1.07 | 2.39 | 2.21E-02 |  |
| 7 | rs556141963 | 80493015 | G | 0.03 | 0.02 |  | 1/63/1729 | 0.04 | 0.04 | 0.4461 |  | | 1.60 | 1.07 | 2.39 | 2.21E-02 |  |
| 10 | rs2227562 | 75672961 | A | 0.23 | 0.29 |  | 152/741/900 | 0.41 | 0.41 | 1.0000 |  | | 0.84 | 0.73 | 0.98 | 2.24E-02 |  |
| 10 | rs2227568 | 75673879 | T | 0.23 | 0.29 |  | 152/741/900 | 0.41 | 0.41 | 1.0000 |  | | 0.84 | 0.73 | 0.98 | 2.24E-02 |  |
| 21 | rs2833326 | 32552630 | A | 0.12 | 0.14 |  | 46/425/1322 | 0.24 | 0.25 | 0.1031 |  | | 0.80 | 0.67 | 0.97 | 2.25E-02 |  |
| 21 | rs62222866 | 32874914 | A | 0.08 | 0.09 |  | 13/314/1466 | 0.18 | 0.17 | 0.4906 |  | | 0.77 | 0.61 | 0.96 | 2.31E-02 |  |
| 7 | rs5850 | 23314547 | T | 0.15 | 0.14 |  | 33/430/1330 | 0.24 | 0.24 | 0.9210 |  | | 0.80 | 0.66 | 0.97 | 2.35E-02 |  |
| 7 | rs10085838 | 23298415 | A | 0.15 | 0.14 |  | 33/431/1329 | 0.24 | 0.24 | 0.8434 |  | | 0.80 | 0.66 | 0.97 | 2.37E-02 |  |
| 21 | rs73191663 | 32553849 | A | 0.12 | 0.14 |  | 44/427/1322 | 0.24 | 0.25 | 0.1788 |  | | 0.81 | 0.67 | 0.97 | 2.44E-02 |  |
| 21 | rs13049312 | 32817622 | C | 0.13 | 0.13 |  | 29/399/1365 | 0.22 | 0.22 | 1.0000 |  | | 1.24 | 1.03 | 1.51 | 2.48E-02 |  |
| 21 | rs2833329 | 32564585 | C | 0.10 | 0.12 |  | 26/367/1400 | 0.20 | 0.21 | 0.7310 |  | | 0.79 | 0.64 | 0.97 | 2.53E-02 |  |
| 21 | rs2262256 | 32565792 | A | 0.10 | 0.12 |  | 26/367/1400 | 0.20 | 0.21 | 0.7310 |  | | 0.79 | 0.64 | 0.97 | 2.54E-02 |  |
| 5 | rs374097640 | 9230270 | T | 0.34 | 0.34 |  | 235/763/795 | 0.43 | 0.45 | 0.0161 |  | | 0.86 | 0.75 | 0.98 | 2.59E-02 |  |
| 5 | rs559748781 | 142011344 | C | 0.46 | 0.37 |  | 244/823/726 | 0.46 | 0.46 | 0.6472 |  | | 1.17 | 1.02 | 1.33 | 2.60E-02 |  |
| 21 | rs2300338 | 32570085 | A | 0.10 | 0.12 |  | 26/366/1401 | 0.20 | 0.21 | 0.7303 |  | | 0.79 | 0.64 | 0.97 | 2.71E-02 |  |
| 5 | rs71611368 | 9221704 | A | 0.01 | 0.03 |  | 0/92/1701 | 0.05 | 0.05 | 0.6289 |  | | 0.60 | 0.38 | 0.94 | 2.75E-02 |  |
| 5 | rs7724468 | 9096441 | G | 0.03 | 0.05 |  | 4/167/1622 | 0.09 | 0.09 | 1.0000 |  | | 0.69 | 0.50 | 0.96 | 2.84E-02 |  |
| 5 | rs72741939 | 9187086 | C | 0.07 | 0.07 |  | 13/223/1557 | 0.12 | 0.13 | 0.1391 |  | | 1.29 | 1.03 | 1.63 | 2.90E-02 |  |
| 21 | rs2284472 | 32515535 | T | 0.26 | 0.26 |  | 98/722/973 | 0.40 | 0.38 | 0.0157 |  | | 1.18 | 1.02 | 1.38 | 3.04E-02 |  |
| 21 | rs58158751 | 32901454 | A | 0.13 | 0.12 |  | 26/373/1394 | 0.21 | 0.21 | 0.8214 |  | | 1.24 | 1.02 | 1.50 | 3.13E-02 |  |
| 21 | rs138244958 | 32556628 | G | 0.15 | 0.18 |  | 58/535/1200 | 0.30 | 0.30 | 0.9366 |  | | 0.83 | 0.69 | 0.98 | 3.17E-02 |  |
| 5 | rs67227299 | 142077467 | T | 0.49 | 0.49 |  | 430/881/482 | 0.49 | 0.50 | 0.5080 |  | | 0.87 | 0.77 | 0.99 | 3.27E-02 |  |
| 21 | rs945262 | 32822340 | G | 0.13 | 0.13 |  | 30/410/1353 | 0.23 | 0.23 | 1.0000 |  | | 1.23 | 1.02 | 1.48 | 3.29E-02 |  |
| 21 | rs58171285 | 32922963 | T | 0.08 | 0.10 |  | 7/341/1445 | 0.19 | 0.18 | 0.0033 |  | | 0.78 | 0.62 | 0.98 | 3.36E-02 |  |
| 16 | rs1132812 | 30198151 | A | 0.04 | 0.07 |  | 9/243/1541 | 0.14 | 0.14 | 1.0000 |  | | 0.76 | 0.59 | 0.98 | 3.38E-02 |  |
| 21 | rs492322 | 32814799 | G | 0.13 | 0.14 |  | 32/449/1312 | 0.25 | 0.25 | 0.4404 |  | | 1.22 | 1.02 | 1.46 | 3.38E-02 |  |
| 5 | rs1013721 | 9088165 | T | 0.03 | 0.05 |  | 4/165/1624 | 0.09 | 0.09 | 1.0000 |  | | 0.70 | 0.51 | 0.98 | 3.47E-02 |  |
| 21 | rs4817383 | 32523356 | C | 0.26 | 0.24 |  | 94/678/1021 | 0.38 | 0.37 | 0.1971 |  | | 1.18 | 1.01 | 1.38 | 3.50E-02 |  |
| 5 | rs79590031 | 142031431 | C | 0.01 | 0.02 |  | 0/72/1721 | 0.04 | 0.04 | 1.0000 |  | | 0.49 | 0.25 | 0.95 | 3.59E-02 |  |
| 21 | rs4411797 | 32920689 | C | 0.08 | 0.10 |  | 7/340/1446 | 0.19 | 0.18 | 0.0033 |  | | 0.79 | 0.63 | 0.98 | 3.61E-02 |  |
| 21 | rs9975837 | 32918532 | T | 0.20 | 0.21 |  | 65/640/1088 | 0.36 | 0.34 | 0.0141 |  | | 0.84 | 0.71 | 0.99 | 3.64E-02 |  |
| 7 | rs10250602 | 23303701 | C | 0.15 | 0.14 |  | 33/427/1333 | 0.24 | 0.24 | 0.9209 |  | | 0.81 | 0.67 | 0.99 | 3.64E-02 |  |
| 7 | rs2307783 | 23307634 | A | 0.27 | 0.27 |  | 130/714/949 | 0.40 | 0.40 | 0.8115 |  | | 0.86 | 0.74 | 0.99 | 3.74E-02 |  |
| 8 | rs113205468 | 27209586 | A | 0.03 | 0.03 |  | 0/115/1678 | 0.06 | 0.06 | 0.2583 |  | | 1.47 | 1.02 | 2.12 | 3.74E-02 |  |
| 5 | rs7714026 | 9087303 | G | 0.14 | 0.18 |  | 43/548/1202 | 0.31 | 0.29 | 0.0349 |  | | 0.83 | 0.69 | 0.99 | 3.76E-02 |  |
| 10 | rs2227560 | 75672475 | G | 0.46 | 0.48 |  | 410/888/495 | 0.50 | 0.50 | 0.7764 |  | | 0.87 | 0.76 | 0.99 | 3.84E-02 |  |
| 21 | rs67520058 | 32600393 | A | 0.39 | 0.40 |  | 291/861/641 | 0.48 | 0.48 | 0.9608 |  | | 0.87 | 0.76 | 0.99 | 3.84E-02 |  |
| 9 | rs148763762 | 5469886 | T | 0.01 | 0.02 |  | 1/56/1736 | 0.03 | 0.03 | 0.3741 |  | | 0.59 | 0.36 | 0.97 | 3.88E-02 |  |
| 5 | rs1806074 | 9204172 | T | 0.21 | 0.12 |  | 35/353/1405 | 0.20 | 0.21 | 0.0301 |  | | 1.29 | 1.01 | 1.65 | 3.95E-02 |  |
| 21 | rs2833309 | 32527137 | T | 0.20 | 0.22 |  | 105/581/1107 | 0.32 | 0.34 | 0.0161 |  | | 1.17 | 1.01 | 1.36 | 3.97E-02 |  |
| 5 | rs707637 | 9445434 | T | 0.17 | 0.24 |  | 103/638/1052 | 0.36 | 0.36 | 0.6459 |  | | 0.85 | 0.73 | 0.99 | 4.02E-02 |  |
| 21 | rs2833440 | 32911276 | C | 0.08 | 0.10 |  | 9/349/1435 | 0.19 | 0.18 | 0.0098 |  | | 0.80 | 0.64 | 0.99 | 4.05E-02 |  |
| 5 | rs6884865 | 9184081 | C | 0.07 | 0.07 |  | 13/221/1559 | 0.12 | 0.13 | 0.0977 |  | | 1.27 | 1.01 | 1.60 | 4.13E-02 |  |
| 7 | rs28635122 | 23310181 | T | 0.15 | 0.14 |  | 33/425/1335 | 0.24 | 0.24 | 1.0000 |  | | 0.82 | 0.67 | 0.99 | 4.15E-02 |  |
| 10 | rs3752986 | 90772781 | A | 0.44 | 0.47 |  | 392/904/497 | 0.50 | 0.50 | 0.6357 |  | | 1.14 | 1.00 | 1.30 | 4.41E-02 |  |
| 21 | rs9983828 | 32630308 | C | 0.20 | 0.16 |  | 43/503/1247 | 0.28 | 0.27 | 0.3901 |  | | 1.20 | 1.00 | 1.43 | 4.46E-02 |  |
| 21 | rs62222875 | 32924009 | T | 0.08 | 0.10 |  | 7/341/1445 | 0.19 | 0.18 | 0.0033 |  | | 0.79 | 0.63 | 0.99 | 4.47E-02 |  |
| 7 | rs3215357 | 80380832 | G | 0.14 | 0.14 |  | 32/429/1332 | 0.24 | 0.24 | 0.7657 |  | | 1.21 | 1.00 | 1.45 | 4.56E-02 |  |
| 17 | rs3817655 | 34199641 | T | 0.39 | 0.36 |  | 223/830/740 | 0.46 | 0.46 | 0.7184 |  | | 1.15 | 1.00 | 1.31 | 4.61E-02 |  |
| 21 | rs9980878 | 32514657 | C | 0.27 | 0.26 |  | 109/728/956 | 0.41 | 0.39 | 0.0594 |  | | 1.16 | 1.00 | 1.35 | 4.65E-02 |  |
| 21 | rs2211881 | 32597023 | A | 0.39 | 0.40 |  | 286/862/645 | 0.48 | 0.48 | 0.9608 |  | | 0.87 | 0.76 | 1.00 | 4.80E-02 |  |
| 21 | rs3787678 | 32597495 | G | 0.40 | 0.41 |  | 304/880/609 | 0.49 | 0.49 | 0.6618 |  | | 0.87 | 0.77 | 1.00 | 4.83E-02 |  |
| 5 | rs4701833 | 9070463 | A | 0.12 | 0.38 |  | 265/826/702 | 0.46 | 0.47 | 0.3934 |  | | 0.87 | 0.76 | 1.00 | 4.87E-02 |  |
| 21 | rs1029229 | 32817026 | C | 0.23 | 0.22 |  | 89/603/1101 | 0.34 | 0.34 | 0.5794 |  | | 1.17 | 1.00 | 1.37 | 4.94E-02 |  |
| 21 | rs1029230 | 32817040 | G | 0.23 | 0.22 |  | 89/603/1101 | 0.34 | 0.34 | 0.5794 |  | | 1.17 | 1.00 | 1.37 | 4.94E-02 |  |
| 5 | rs250114 | 141999269 | G | 0.08 | 0.25 |  | 126/661/1006 | 0.37 | 0.38 | 0.2369 |  | | 0.86 | 0.74 | 1.00 | 4.95E-02 |  |
| Note: NSCLO, non-syndromic cleft lip only; SNP, Single Nucleotide Polymorphism; A1, minor allele; MAF, minor allele frequency; HWE, hardy-Weinberg equilibrium; GENO, Genotype counts; O(HET), Observed heterozygosity; E(HET), Expected heterozygosity; P_HWE_, Hardy-Weinberg test P value; OR, odds ratio; 95%CI, 95% confidence level; L95, lower limit of the 95%CI; U95, upper limit of the 95%CI; P, P value for association analysis. | | | | | | | | | | | | | | | | |  |

| **Supplementary Table 7 Annotation for NSCLO associated SNPs by using of Haploreg v4.1** | | | | | | | | | | | | | | | |  |
| --- | --- | --- | --- | --- | --- | --- | --- | --- | --- | --- | --- | --- | --- | --- | --- | --- |
| **SNP** | **Motifs changed^a^** | **GENCODE genes** | **Function annotation** | **Epigenome ID (EID)** | **Group** | **Description** | **Chromatin states (Core 15-state model) ^b^** | **Chromatin states (25-state model using 12 imputed marks) ^b^** | **H3K4me1^b^** | **H3K4me3^b^** | **H3K27ac^b^** | **H3K9ac^b^** | **PMID^c^** | **Tissue^c^** | **Correlated gene^c^** |  |
|  |  |  |  |  |  |  |  |  |  |  |  |  |  |  |  |  |
| [rs201795193](https://pubs.broadinstitute.org/mammals/haploreg/detail_v4.php?query=&id=rs201795193) | Dobox4 | THADA | intronic | E127 | ENCODE2012 | NHEK |  |  |  |  |  |  |  |  |  |  |
|  | Hlx1 |  |  |  |  |  |  |  |  |  |  |  |  |  |  |  |
|  | Ik-2_3 |  |  |  |  |  |  |  |  |  |  |  |  |  |  |  |
|  | Pax7 |  |  |  |  |  |  |  |  |  |  |  |  |  |  |  |
|  | Pou1f1_2 |  |  |  |  |  |  |  |  |  |  |  |  |  |  |  |
|  | Pou3f2_4 |  |  |  |  |  |  |  |  |  |  |  |  |  |  |  |
| [rs12990267](https://pubs.broadinstitute.org/mammals/haploreg/detail_v4.php?query=&id=rs12990267) |  | THADA | intronic |  |  |  |  |  |  |  |  |  |  |  |  |  |
| [rs199721109](https://pubs.broadinstitute.org/mammals/haploreg/detail_v4.php?query=&id=rs199721109) | Nkx6-1 | THADA | intronic |  |  |  |  |  |  |  |  |  |  |  |  |  |
| [rs74343467](https://pubs.broadinstitute.org/mammals/haploreg/detail_v4.php?query=&id=rs74343467) | E2F | THADA | intronic |  |  |  |  |  |  |  |  |  |  |  |  |  |
|  | Irf |  |  |  |  |  |  |  |  |  |  |  |  |  |  |  |
| [rs6544660](https://pubs.broadinstitute.org/mammals/haploreg/detail_v4.php?query=&id=rs6544660) | Gfi1b | THADA | intronic | E127 | ENCODE2012 | NHEK | 7_Enh | 14_EnhA2 | H3K4me1_ Enh |  | H3K27ac_ Enh | H3K9ac_ Pro | [24013639](http://pubmed.gov/24013639) | Whole_ Blood | ZFP36L2 |  |
|  | Nkx2 |  |  |  |  |  |  |  |  |  |  |  | [22685416](http://pubmed.gov/22685416) | Cerebellum | THADA |  |
|  |  |  |  |  |  |  |  |  |  |  |  |  | [22685416](http://pubmed.gov/22685416) | Temporal_ Cortex | THADA |  |
| [rs12478601](https://pubs.broadinstitute.org/mammals/haploreg/detail_v4.php?query=&id=rs12478601) | Hoxb13 | THADA | intronic | E127 | ENCODE2012 | NHEK |  |  | H3K4me1_Enh |  |  |  | [24604202](http://pubmed.gov/24604202) | Monocytes_Naive | THADA |  |
|  | Hoxd10 |  |  |  |  |  |  |  |  |  |  |  | [24604202](http://pubmed.gov/24604202) | Monocytes_Naive | ZFP36L2 |  |
|  | LXR |  |  |  |  |  |  |  |  |  |  |  | [22685416](http://pubmed.gov/22685416) | Cerebellum | THADA |  |
|  |  |  |  |  |  |  |  |  |  |  |  |  | [22685416](http://pubmed.gov/22685416) | Temporal_ Cortex | THADA |  |
| **Note:** chr, chromosome; pos, position; SNP, Single Nucleotide Polymorphism; Ref, reference allele; Alt, alternate allele. **^a^**Transcription factor binding and regulation motifs; **^b^**Regulatory chromatin states from DNAse and histone ChIP-Seq (Roadmap Epigenomics Consortium, 2015); H3K4me1 and H3K27ac for enhancer, H3K9ac for promoter in NHEK(Epidermal Keratinocyte Primary Cells) .**^c^**eQTL studies showing correlation of SNP with cis expression. | | | | | | | | | | | | | | | |  |
